# Monoamine oxidase A (MAO A) inhibitors decrease glioma progression

**DOI:** 10.18632/oncotarget.7283

**Published:** 2016-02-09

**Authors:** Swati Kushal, Weijun Wang, Vijaya Pooja Vaikari, Rajesh Kota, Kevin Chen, Tzu-Shao Yeh, Niyati Jhaveri, Susan L. Groshen, Bogdan Z. Olenyuk, Thomas C. Chen, Florence M. Hofman, Jean C. Shih

**Affiliations:** ^1^ Department of Pharmacology and Pharmaceutical Sciences, School of Pharmacy, University of Southern California, Los Angeles, California, USA; ^2^ Department of Neurosurgery, Keck School of Medicine, University of Southern California, Los Angeles, California, USA; ^3^ Department of Pathology, Keck School of Medicine, University of Southern California, Los Angeles, California, USA; ^4^ Department of Cell and Neurobiology, Keck School of Medicine, University of Southern California, Los Angeles, California, USA; ^5^ USC-Taiwan Center for Translational Research, Los Angeles, California, USA; ^6^ Program for Cancer Biology and Drug Discovery, College of Pharmacy, Taipei Medical University, Taipei, Taiwan; ^7^ Preventive Medicine, Keck School of Medicine, University of Southern California, Los Angeles, California, USA

**Keywords:** MAO A, MAO A inhibitors, glioma, TMZ-resistant, near-infrared dye conjugate

## Abstract

Glioblastoma (GBM) is an aggressive brain tumor which is currently treated with temozolomide (TMZ). Tumors usually become resistant to TMZ and recur; no effective therapy is then available. Monoamine Oxidase A (MAO A) oxidizes monoamine neurotransmitters resulting in reactive oxygen species which cause cancer. This study shows that MAO A expression is increased in human glioma tissues and cell lines. MAO A inhibitors, clorgyline or the near-infrared-dye MHI-148 conjugated to clorgyline (NMI), were cytotoxic for glioma and decreased invasion *in vitro*. Using the intracranial TMZ-resistant glioma model, clorgyline or NMI alone or in combination with low-dose TMZ reduced tumor growth and increased animal survival. NMI was localized specifically to the tumor. Immunocytochemistry studies showed that the MAO A inhibitor reduced proliferation, microvessel density and invasion, and increased macrophage infiltration. In conclusion, we have identified MAO A inhibitors as potential novel stand-alone drugs or as combination therapy with low dose TMZ for drug-resistant gliomas. NMI can also be used as a non-invasive imaging tool. Thus has a dual function for both therapy and diagnosis.

## INTRODUCTION

Glioblastoma (GBM) is the most aggressive form of primary brain tumors, with a median survival time of 14 months from the time of diagnosis [1]. Temozolomide (TMZ) is the current therapeutic agent for treating newly diagnosed GBM either in combination with surgery and radiation or as stand-alone chemotherapy [2]. Unfortunately, following TMZ treatment, tumors recur; and these tumors are TMZ-resistant. Increasing TMZ doses is not an option because this DNA alkylating agent is highly toxic to the bone marrow [3]. At this point, therapy choices are very limited. Therefore, identifying drugs that can cross the blood-brain- barrier (BBB), and are effective in decreasing the tumor progression of TMZ-resistant gliomas is critical.

MAO A and MAO B are two isoenzymes encoded by two different genes [4]. They have 70 % amino acid identity with different substrates and inhibitor specificity [5]. MAO A inhibitors have been shown to cross the BBB, as noted by their use in the treatment of various neuropsychiatric disorders [6]. The preferred substrates for MAO A are serotonin, norepinephrine and dopamine. MAO A can be inhibited by low concentrations of clorgyline. By contrast, MAO B preferred substrates are phenylethylamine and benzylamine, and MAO B can be inhibited by low dose deprenyl [7]. This is the first study to show that MAO A inhibitors may be used alone or in combination with low dose TMZ to reduce tumor progression and increase survival, a potential treatment for TMZ-resitant, recurrent gliomas.

## RESULTS

### MAO A is expressed in human glioma tissues and cells

**Figure 1:**
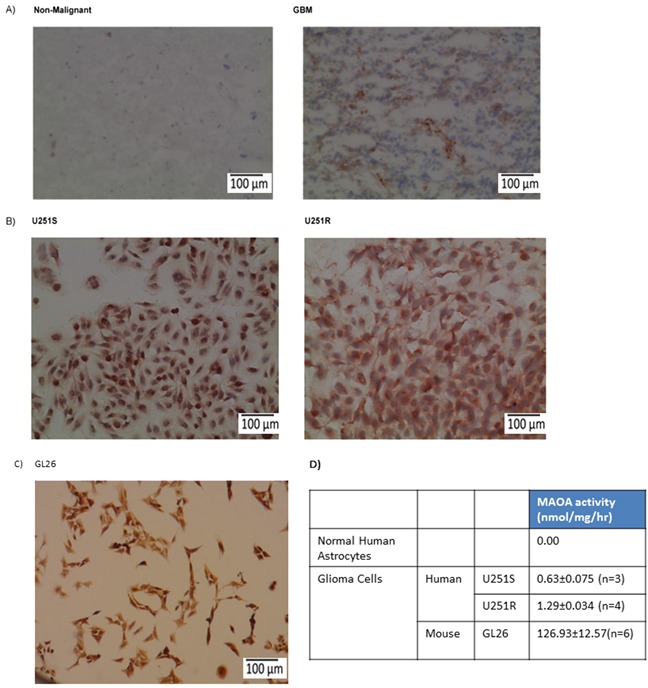
MAO A expression and activity are increased in mouse and human glioma cell lines and glioma tissues **A.** Non-malignant brain and glioma (GBM) tissue specimens, **B.** U251S (TMZ-sensitive) and U251R (TMZ-resistant) human glioma cells, and **C.** GL26 mouse glioma cells were stained for MAO A. The red precipitate denotes positive staining. Scale bars represent 100 μm. **D.** MAO A catalytic activity in U251S, U251R, GL26, and normal astrocytes was determined.

Fresh frozen human glioma tissues were analyzed for MAO A expression; as a control, non-malignant brain tissues were examined in parallel. Our results show significant expression of MAO A in GBM tissues but no detectable staining in non-malignant brain tissue (Figure 1A). Based on morphology, the staining in GBM is associated with tumor cells. Both TMZ-sensitive (U251S) and TMZ-resistant (U251R) (Figure 1B) human glioma cells and mouse glioma GL26 cells (Figure 1C) expressed MAO A. MAO A catalytic activity was determined in human and mouse glioma cell lines using serotonin as the substrate (Figure 1D). We found that both human and mouse glioma cells expressed MAO A catalytic activity. By contrast, normal human astrocytes exhibited no detectable MAO A activity (Figure 1D) consistent with the immunostaining results of non-malignant brain tissue (Figure 1A).

### The MAO A inhibitor, clorgyline, alone or with low dose TMZ induced cytotoxicity in TMZ-sensitive glioma cells *in vitro*

**Figure 2:**
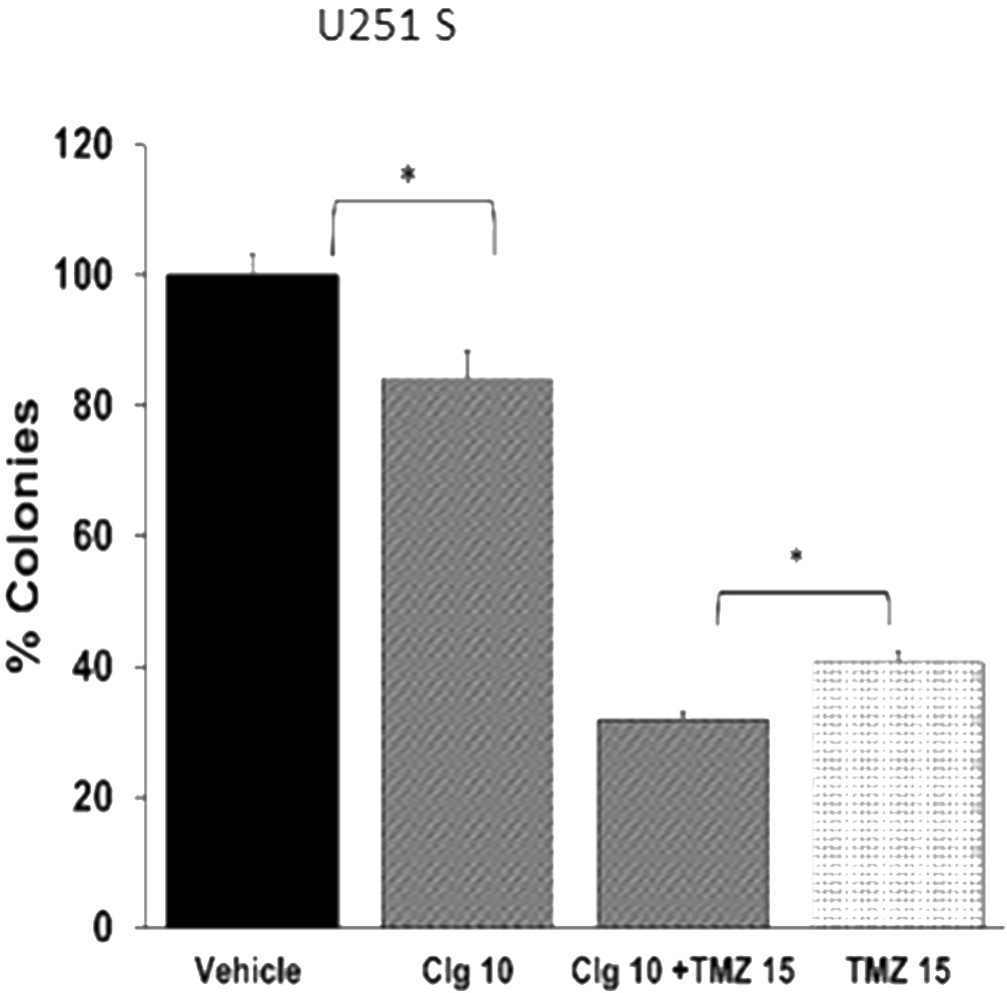
Clorgyline enhances the cytotoxic efficacy of TMZ-sensitive glioma cells *in vitro* **A.** U251S cells were treated with clorgyline (10 μM) alone or combination with TMZ (15 μM) for 48 hours, then incubated for another 10 days for the colony forming assay (CFA). Colonies were stained and quantified. Vehicle-treated cells were considered to be 100%. Clorgyline increased cytotoxicity (10 μM) (*p<0.05). The addition of clorgyline significantly increased the efficacy of TMZ (*p<0.05).

To study the effects of clorgyline on TMZ-sensitive human glioma cells, TMZ-sensitive glioma cells, U251S, were treated with the MAO A inhibitor clorgyline alone or in combination with TMZ. Treatment of cells with clorgyline (10 μM) alone increased cytotoxicity in U251S cells by 20% (*p<0.05) (Figure 2). TMZ (15 μM) alone increased cytotoxicity to 60% (*p<0.05). Combined treatment of clorgyline with TMZ increased cytotoxicity to 70% (*p<0.05) (Figure 2). These results demonstrate that clorgyline can significantly increase the efficacy of TMZ (*p<0.05). Thus, the MAO A inhibitor enhances the effect of TMZ on TMZ-sensitive glioma cells. Clorgyline alone or in combination with TMZ did not have an effect on cytotoxicity of TMZ-resistant GBM cells (data not shown).

### Near–infrared dye conjugated MAO A inhibitor clorgyline (NMI) localizes to the mitochondria of cancer cells

**Figure 3:**
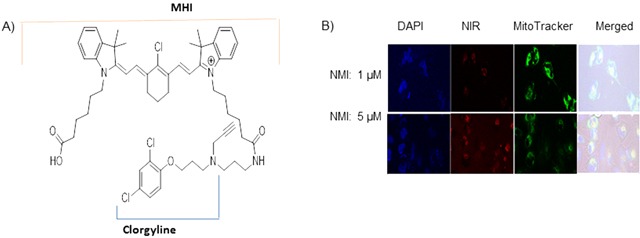
Near–infrared dye conjugated to MAO A Inhibitor clorgyline (NMI) is localized to the mitochondria of cancer cells **A.** The structure of NMI is composed of clorgyline conjugated to MHI-148 dye. **B.** NMI (red color) is localized in the mitochondria. Glioma cells U251 S or R were treated with NMI (1, 5 μM) for 3 hours; MitoTracker reagent stained the mitochondria green. Merged images demonstrated red dye localized to green mitochondria.

In order to deliver MAO A specifically to GBM cells, we conjugated tumor-specific near-infrared dye (NIR), MHI-148, to the MAO A inhibitor, clorgyline, to generate a novel chemical compound, NMI. This drug (Figure 3A) preferentially accumulates in cancer lesions [8]. Synthesis of NMI is detailed in Supplementary Figure S1.

To evaluate the cellular uptake of NMI in human glioma cells, laser-scanning confocal microscopy was utilized. The confocal-microscopic images of tumor cells treated with NMI (1μM or 5μM) are shown (Figure 3B). This compound rapidly accumulated in U251 S or R glioma cells and co-localized with the mitochondria-specific dye, MitoTracker Green (Figure 3B). This suggests that NMI is localized in the mitochondria as expected. The inhibitory effects of NMI on MAO A activity were determined in GL26 mouse glioma cells. We found that NMI inhibited MAO A activity at low doses with an IC_50_ of 5 × 10^−6^. These results indicate that NMI is specifically targeted to glioma cells mitochondria and inhibit MAO A activity *in vitro*.

### NMI decreases the viability of TMZ-sensitive and -resistant glioma cells *in vitro*

**Figure 4:**
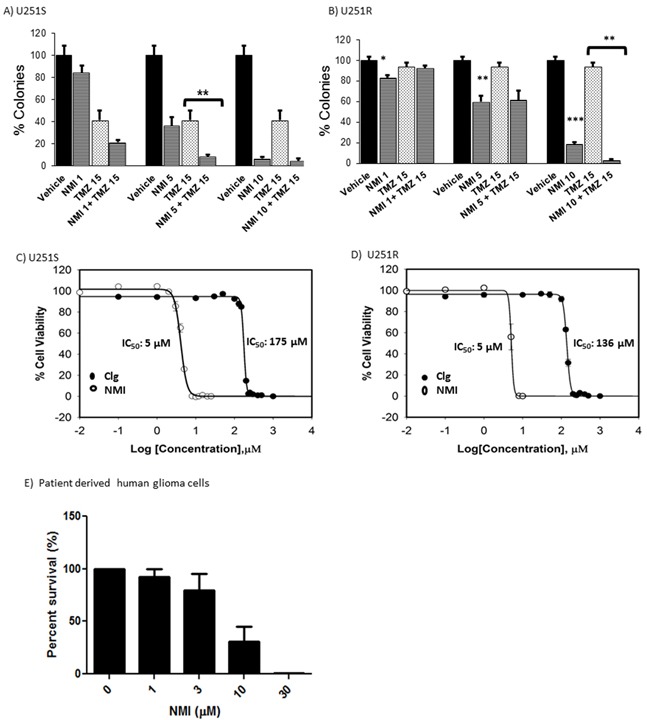
Cytotoxic effects of NMI on human glioma cells **A.** Cytotoxicity was evaluated for U251S and **B.** U251R glioma cells treated with vehicle, TMZ (15 μM) and NMI alone (1, 5, 10 μM), or in combination with TMZ for 48 hours; colonies were counted and normalized to vehicle (100%). **C.** Effects of clorgyline and NMI on cell viability in U251S and **D.** U251R human glioma cells as measured by the MTS assay. **E.** Patient-derived glioma cells were treated with NMI at 1, 3, 10 and 30 μM for 96 hours, then incubated for another 7 days for colony forming assay (CFA). Colonies in experimental groups were normalized to control (no treatment) and set at 100%. Error bars = standard error of the mean (SEM); experiments were performed in triplicate. Significance was determined using the Student's t-test: *** *p* < 0.0001, ** *p* < 0.005, * *p* <0.05.

Glioma recurrence is associated with acquiring TMZ resistance; therefore, we investigated whether NMI is cytotoxic to TMZ-resistant (U251R) glioma cells. Glioma cells were treated with NMI at doses ranging from 1-10 μM. The results show that treatment of U251S cells with NMI alone at 5 μM and 10 μM significantly reduced colony formation by 60% and 90%, respectively (Figure 4A) The addition of TMZ (15 uM) to NMI significantly increased cytotoxicity, especially at 5uM of NMI (Figure 4A). These results indicate that NMI can enhance the cytotoxicity of TMZ.

We then evaluated the effects of NMI on TMZ-resistant human glioma cells (Figure 4B). TMZ alone (15 μM) had no effect as expected. In contrast, NMI exhibited a dose-dependent increase in cytotoxicity at 1, 5 and 10 μM by 20%, 40% and 80%, respectively compared to the vehicle (Figure 4B). NMI at 10 μM in combination with TMZ caused a further significant increase in cell death. These results showed that in TMZ-resistant cells, NMI is effective as a monotherapy or in combination with TMZ. Combining NMI with TMZ was more effective for therapy than either drug alone.

The cytotoxic effects of clorgyline and NMI on glioma cells were studied using the MTS assay (Figure 4C and 4D). Treatment with clorgyline yielded dose response curves with 50% inhibitory concentrations (IC_50_) of approximately 175 μM and 136 μM in U251S and U251R cells, respectively. In contrast, NMI was cytotoxic at an IC_50_ of 5 μM in both cell lines, indicative of 30 to 35-fold higher efficacy as compared to clorgyline. The cytotoxic effect of NMI was also examined using patient-derived glioma cells. NMI (10μM) exhibited approximately 70% cytotoxicity (*p<0.05) (Figure 4E). Clorgyline at these low concentrations had no effect on these cells (data not shown). These data demonstrate that in both TMZ-sensitive and resistant glioma cells, and patient-derived cells, NMI is a significantly more effective cytotoxic agent than clorgyline.

### NMI inhibits invasion of patient-derived glioma cells

**Figure 5:**
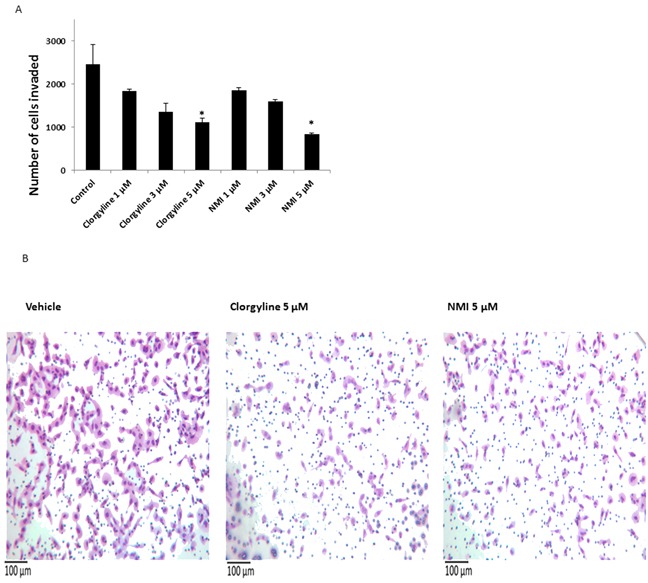
The effects of clorgyline and NMI on the invasive capacity of glioma cells **A.** Patient -derived glioma cells were seeded in invasion chambers, treated with clorgyline (1, 3, 5 μM) and NMI (1, 3, 5 μM) for 22 hours, and then analyzed for the number of cells that invaded to the underside of the filter. **B.** Representative images (200X) of vehicle, clorgyline (5 μM) and NMI (5 μM). Scale bar represents 100 μm for all figures.

The effects of NMI on the invasion of patient-derived glioma cells were tested in a Boyden chamber invasion assay. The results showed that NMI (5 μM) significantly reduce glioma cell invasion (*p<0.05) (Figure 5A). Clorgyline at 5μM was also effective at decreasing invasion. Representative images of invasion assay showing a decrease in cell number following the treatment with NMI (5 μM) or clorgyline (5μM) are shown (Figure 5B).

### NMI specifically targets cancer cells *in vivo*

**Figure 6:**
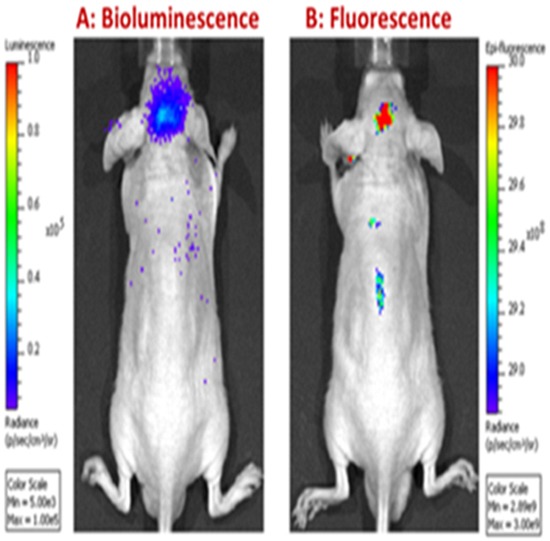
NMI specifically targets cancer cells Animals were implanted with luciferase-labeled glioma cells. After 10 days, NMI was administered by subcutaneous injection and imaged for **A.** bioluminescence and **B.** fluorescence. Bioluminescence images identified brain tumor sites with luciferase; fluorescence identified the sites of NMI localization.

Near infrared (NIR) fluorescence imaging of tumor-bearing animals treated with NMI showed specific uptake and accumulation of NMI in glioma cells. The bioluminescence of luciferase- labeled cells as well as the fluorescence of NMI was recorded after 10 days of daily subcutaneous injection of NMI. Overlaying the NIR image with bioluminescence showed that NMI localized to the tumor site with no detectable distribution to other organs (Figure 6).

### Clorgyline or NMI alone or in combination with TMZ increases survival *in vivo*

**Figure 7:**
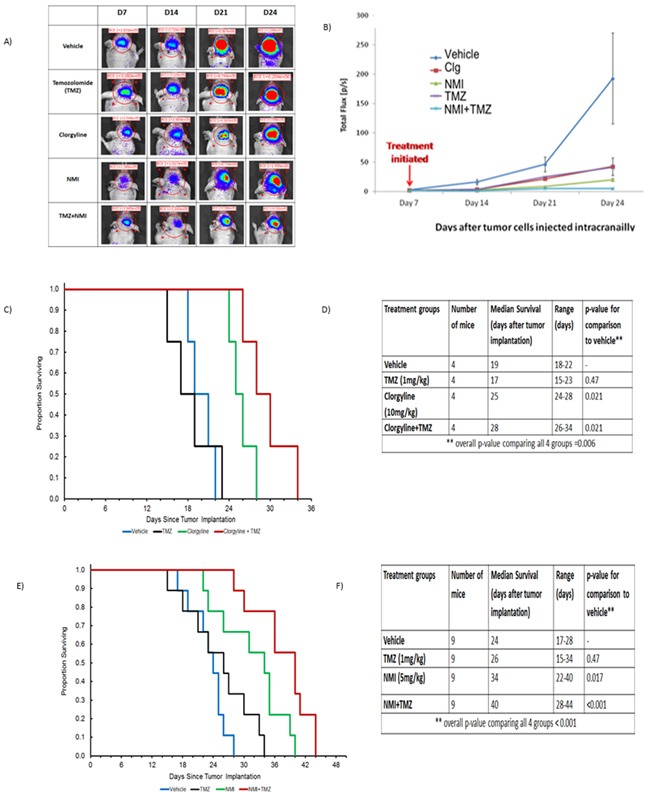
Clorgyline, NMI or the combination with TMZ reduces tumor progression and increases the survival of TMZ-resistant tumors Athymic/nude mice were implanted intracranially with TMZ-resistant human glioma cells (U251R). Animals were then treated daily with: vehicle, TMZ (1 mg/kg), clorgyline (10 mg/kg), TMZ (1 mg/kg) + clorgyline (10 mg/kg), NMI (5 mg/kg), or TMZ (1 mg/kg) +NMI (5 mg/kg). MAO A inhibitors were administered subcutaneously daily for 21 days; TMZ treatment was given for the first 10 days only. **A.** Animals were imaged on days 7, 14, 21, 24 post-implantation. **B.** Graphic representation of bioluminescence imaging conducted at successive time points. **C.** The Kaplan-Meier survival curve shows that clorgyline significantly prolonged survival. **E.** The Kaplan-Meier survival curve for NMI and NMI+TMZ shows that NMI was effective alone, as compared to the vehicle, and the combination of NMI and TMZ was significantly more effective (*p < 0.001).

To determine the *in vivo efficacy of* MAO A inhibitors on TMZ-resistant tumors U251R cells were implanted intracranially into nude mice. Animals were imaged 7 days post implantation and grouped. Mice (n=4) were treated with: clorgyline (10 mg/kg), NMI (5 mg/kg), TMZ (1 mg/kg) alone or in combination with TMZ. Clorgyline or NMI were administered subcutaneously daily for 21 days; TMZ was administered orally for the first 10 days (1 mg/kg). This low dose of TMZ was used to identify any additive effects of TMZ to clorgyline or NMI. Animals were imaged on days 7, 14, 21, 24 (Figure 7A) post tumor implantation. After 28 days (i.e. 7 days after implantation and 21 days post treatment), treatment was stopped; tumor growth (Figure 7B) and survival (Figure 7C) were documented. Median survival data (Figure 7D) of the vehicle group and TMZ-treated group were not significantly different (*p*=0.47). However, tumor progression was delayed in the clorgyline treated group compared to the vehicle (*p < 0.05), (Figures 7C and 7D). Furthermore, addition of clorgyline to the low dose TMZ enhanced the effects of TMZ (Clorgyline + TMZ versus TMZ alone) (*p< 0.05).

Studies utilizing NMI showed that treatment with NMI alone increased median survival as compared to vehicle (*p=0.017) (Figure 7E and 7F). The addition of NMI to TMZ further increased the median survival (*p < 0.001). Furthermore, NMI enhanced the effects of low dose TMZ. No significant changes in body weight were observed with drug treatment. These data indicate that clorgyline and NMI delay tumor progression, and NMI or clorgyline in combination with TMZ further increases survival time. Thus, combining MAO A inhibitors with low dose TMZ can enhance the therapeutic efficacy of TMZ.

### Clorgyline and NMI reduce proliferation and angiogenesis of glioma, and increase macrophage infiltration in tumors *in situ*

**Figure 8:**
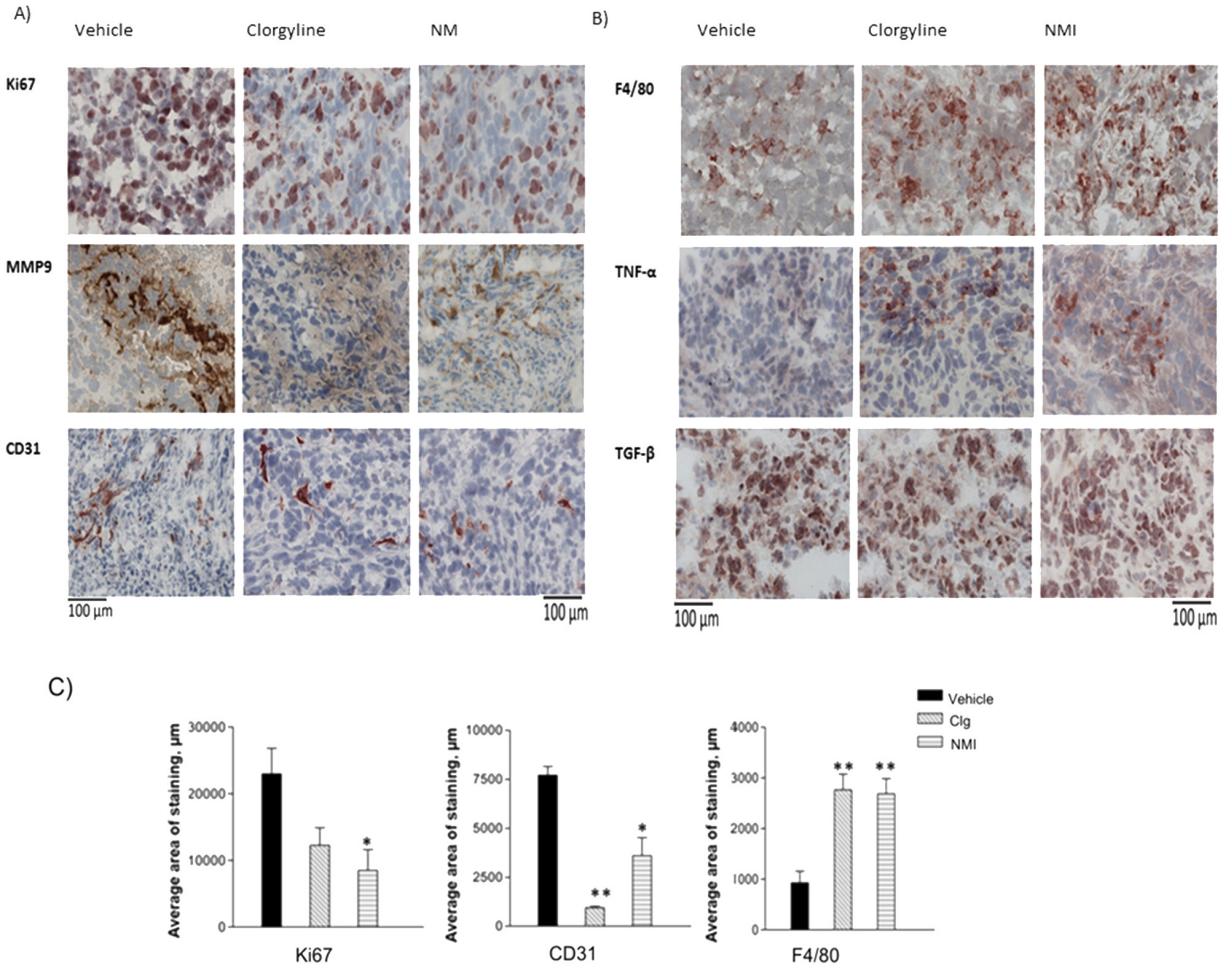
Clorgyline and NMI reduce proliferation, invasion of glioma and microvessel density, as well as enhance macrophage accumulation *in vivo* **A.** Tumor tissues from the different groups were immunostained for: proliferation (Ki67), invasion (matrix metalloproteinase 9 (MMP)) and microvessel density (CD31). **B.** Tumor tissue sections were immunostained for macrophages (F4/80), tumor necrosis factor (TNF) -α and transforming growth factor (TGF)-β. **C.** Immunostaining was analyzed using the ImageJ software; *p* values were calculated compared to the vehicle. Tissues from clorgyline-treated and NMI-treated mice exhibited reduced microvessel density (**p < 0.01, *p < 0.05) respectively, and increased macrophages (**p < 0.01), as well as reduced proliferation (*p < 0.05) in NMI only. Scale bar represent 100 μm for all figures. The red color indicates positive staining (mag. 400x).

To identify potential mechanisms for increased survival, tumor tissues were analyzed for proliferation, microvessel density (MVD), inflammatory cell infiltration, and secretion of growth factors using immunostaining techniques. Cell proliferation was identified using, Ki67. The results show that the number of Ki67 positive cells decreased in clorgyline and NMI-treated animals (*p* < 0.05), as compared to the vehicle (Figure 8A, row 1). Tissues were analyzed for matrix metalloproteinase 9 (MMP9), an enzyme responsible for the breakdown of extracellular matrix and increase in tumor invasion [9]. The results (Figure 8A, row 2) indicate increased MMP9 in vehicle treated tumor tissues as compared to clorgyline or NMI-treated animals. These data suggest that MAO A inhibitors reduced tumor cell invasiveness. Angiogenesis was assessed by staining for CD31, an endothelial cell marker. The results (Figure 8A, row 3) showed that clorgyline (*p* < 0.01) and NMI (*p* < 0.05)-treated animals had significantly reduced MVD compared to the vehicle group. These data demonstrated that clorgyline and NMI reduced proliferation, invasion and angiogenesis in tumors, thereby contributing to the enhanced survival.

The innate immune response is an important modulator of tumor growth [10]. We therefore analyzed inflammatory cells in tumor tissues using the macrophage marker F4/80 (Figure 8B, row 1). The results show a significant increase in the number of macrophages in MAO A inhibitor-treated animals as compared to vehicle-treated animals (*p*< 0.01). Inflammatory cytokines are responsible for much of the activity attributed to macrophages. To determine whether the macrophages detected in tumor tissues were proinflammatory, tissue specimens were examined for tumor necrosis factor (TNF)-α, a powerful proinflammatory growth factor [11]. Results demonstrated an increased TNF-α positive population in tumors from animals treated with the MAO A inhibitor (Figure 8B, row 2), suggesting that MAO A inhibitors upregulate the proinflammatory response and decreased tumor progression. The expression of transforming growth factor (TGF β), an immune regulatory growth factor [12], was not affected by MAO A inhibitors (Figure 8B, row 3). Quantitation of Ki67, CD31and F4/80 staining results is presented (Figure 8C). These data provide compelling evidence that MAO A inhibitors reduce glioma growth *in vivo* by increasing tumor cell death, and decreasing tumor invasiveness.

## DISCUSSION

The data presented here demonstrate that the MAO A inhibitors, clorgyline and its conjugate NMI, are effective in reducing TMZ-resistant tumor growth and increasing survival in glioma. This is consistent with our previous report that knock-down (KD) or pharmacological inhibition of MAO A in prostate cancer reduced or eliminated cancer progression [13, 14].

MAO A expression is increased in human glioma tissue specimens and cells. Therefore we studied the effects of the MAO A inhibitor, clorgyline, and NMI, the conjugate of clorgyline with near-infrared-dye MHI-148. These agents cross the BBB; and NMI specifically targets mitochondria in human glioma cells. Our data shows that NMI have better efficacy than clorgyline in both *in vitro* and *in vivo* studies. This may be due to the fact that NMI binds specifically to the cancer cells. *In vitro* studies showed that NMI was cytotoxic to TMZ-sensitive and -resistant glioma cells, and to patient–derived tumor cells. These cells were determined to be MGMT-positive, a common cause of TMZ resistance [15]. The dose of TMZ used *in vivo* was 1 mg/kg, which is approximately 25 times less than the doses administered to patients [2]. Thus MAO A inhibitors can enhance the efficacy of relatively non-toxic doses of TMZ, thereby diminishing the secondary effects of TMZ, such as myelosuppresion.

The potential mechanisms underlying the *in vivo* effects of MAO A inhibitors were studied using tumor tissues from clorgyline and NMI treated mice. The data indicated that both clorgyline and NMI decreased blood vessel growth (Figure 8). Clorgyline and NMI do not appear to affect normal blood vessels since the blood vessel density in the adjacent brain parenchyma showed no abnormal density or vascular structure (data not shown). Thus, MAO A inhibitors specifically affect the tumor vasculature and not normal brain endothelial cells. The microenvironment of the tumor vasculature also expresses high levels of vascular endothelial growth factor (VEGF), and basic fibroblast growth factor (bFGF), as well as low levels of thrombospondin-1 (TSP-1), compared to normal brain [16, 17]. We are currently investigating whether MAO A inhibitors regulate the secretion of these growth factors.

Tumor tissues from mice treated with clorgyline or NMI exhibited high numbers of macrophages and a significant increase in TNF-α expression, indicating that the macrophages present were likely to be proinflammatory cells, thus invovled in reduced tumor progression.

In conclusion, we have shown that MAO A inhibitors reduce TMZ-resistant glioma progression, thereby increasing survival time. The effects of MAO A inhibitors may be the result of increased cytotoxicity, and/or decreased invasion of tumor cells. MAO inhibitors in combination with low dose TMZ increase the efficacy of TMZ without the toxic effects of high dose TMZ. Thus inhibiting MAO A may be an effective approach to the treatment of recurrent brain tumors. In addition to the therapeutic effects of NMI, this agent can also be used for non-invasive diagnosis and monitoring of tumor progression.

## MATERIALS AND METHODS

### Tissues and cell cultures

Human glioma cell line U251 was obtained from American Type Culture Collection (ATCC); TMZ-resistant human glioma cells, U251R, were derived as previously described [18]. The patient- derived glioma cells were obtained from our laboratory (FMH, TCC). Glioma cell lines were cultured in 10% fetal calf serum in Dulbecco's Modified Eagle's Media (Life technologies, Carlsbad, CA) supplemented with 100 U/mL penicillin and 0.1 mg/mL streptomycin in a humidified incubator at 37°C and 5% CO_2_. Patient-derived cells were cultured in serum-free media. Human glioma cell lines were authenticated, and tested negative for mycoplasm. Freshly resected brain tissues were obtained and processed in accordance with the USC Institutional Review Board guidelines. Non-malignant brain tissue was obtained following epilepsy surgery. These tissues were snap- frozen and stored at −80°C.

### MAO A catalytic activity assay

MAO A catalytic activity was determined by radioassay as described previously [19]. Briefly cells were incubated with 1 mM ^14^C-5-hydroxytryptamine (5-HT), in the assay buffer. The reaction products were extracted and the radioactivity was determined by liquid scintillation spectroscopy.

For inhibition activity assay, cells were pre-incubated with various compounds at increasing concentrations for 20 minutes at 37°C followed by the addition of ^14^C labelled 5-HT at 37°C for 20 minutes.

### Laser-scanning confocal microscopy

Human glioma cells (U251S or U251R) were plated in glass bottom microscopy dishes (MatTek) (30,000 cells/400 μl), in standard medium, for 24 hours and treated with NMI (1 and 5 μM), Mitotracker Green (200 nM) (Life Technologies, Carlsbad, CA) and DAPI (1x); and incubated for 3 hours. DAPI and Mitotracker agent were used to stain the nucleus and mitochondria respectively. Imaging was performed on a Zeiss LSM 510 inverted laser-scanning confocal microscope. Excitation wavelengths were set at λ_max_ =790 nm Chameleon (DAPI, blue excitation), 488 nm (Mitotracker Green, green-yellow excitation) and 633 nm (NMI, red excitation). The data were acquired in a multi-track mode. Images were taken using pinholes of 130-200 μM.

### Colony forming assay

U251S and U251R glioma cells were seeded in 6-well plates in triplicates at 300 and 400 cells per well respectively and allowed to adhere overnight. Subsequently, cells were treated with clorgyline, NMI and TMZ at various concentrations for 48 hours; the medium was then removed and fresh medium (without drugs) was added. Cells were incubated for an additional 8 to 10 days; colonies were visualized by staining with 1% methylene blue in methanol for 4 hours and quantified.

### MTS assay

Glioma cells were seeded in quadruplicates; clorgyline and NMI were added for 48 hours. Viability was determined as per manufacturer's instructions (Promega, Madison, WI); and calculated relative to untreated control cells. Data was plotted using SigmaPlot 12.0.

### Invasion assay

Cells were seeded in 8 μM matrigel coated invasion chambers (Corning, Corning, NY) with NMI or clorgyline, for 22 hours. Subsequently invaded cells were stained using the hemacolor kit (EMD Millipore, Massachusetts) and imaged. Stained cells were counted manually for analysis.

### *In vivo* studies

All animal protocols were approved by the Institutional Animal Care and Use Committee (IACUC) of USC. For the xenograft model, 4- to 6-week-old male athymic nude mice were purchased from Harlan (Indiana, IN USA). 2 × 10^5^ luciferase-positive TMZ-resistant human glioma cells (U251R) were injected intracranially. Mice were imaged 7 days after implantation. Once tumors appeared in all mice, the animals were grouped into different treatment groups. Clorgyline was dissolved in water, NMI was diluted in vehicle (10% DMSO+ 45% ethanol + 45% glycerol) and administered by subcutaneous injection in a volume of 30 μl, TMZ (1 mg/kg) was diluted in water and administered as gavage. Combination of TMZ and clorgyline or NMI was given at the same dose. NMI, clorgyline and vehicle were administered daily for 21 days; TMZ was administered for the first 10 days only.

### Immunohistochemistry (IHC)

Frozen tissues or cells were fixed in acetone. IHC was performed as described previously [20]. The following antibodies were used: F4/80, TGF-β, TNF-α (Abcam, Cambridge, MA), Ki67 (Santa Cruz Biotechnology Inc., Santa Cruz, CA), CD31 (BD Biosciences, San Jose, CA), MMP 9, MAO A (Santa Cruz Biotechnology Inc., Santa Cruz) as well as biotinylated secondary antibodies (Vector Laboratories, Burlingame, CA). Images were analyzed using ImageJ software.

### Statistical analysis

For *in vivo* experiments, all mice were followed until death; therefore the Kruskal-Wallis test was used to compare all treatment groups. For Figure 7, with more than 2 groups of mice, the overall p-value (comparing all groups) was less than 0.05 therefore pairwise comparisons were made comparing each of the active treatments to the control. All p-values were reported as two-sided; no adjustments were made for multiple comparisons

## SUPPLEMENTARY MATERIAL AND FIGURE



## References

[1] Weis SM, Cheresh DA (2011). Tumor angiogenesis: molecular pathways and therapeutic targets. Nat Med.

[2] Stupp R, Mason WP, van den Bent MJ, Weller M, Fisher B, Taphoorn MJ, Belanger K, Brandes AA, Marosi C, Bogdahn U, Curschmann J, Janzer RC, Ludwin SK (2005). Radiotherapy plus concomitant and adjuvant temozolomide for glioblastoma. N Engl J Med.

[3] Sorrentino BP (2002). Gene therapy to protect haematopoietic cells from cytotoxic cancer drugs. Nat Rev Cancer.

[4] Bach AW, Lan NC, Johnson DL, Abell CW, Bembenek ME, Kwan SW, Seeburg PH, Shih JC (1988). cDNA cloning of human liver monoamine oxidase A and B: molecular basis of differences in enzymatic properties. Proc Natl Acad Sci U S A.

[5] Grimsby J, Chen K, Wang LJ, Lan NC, Shih JC (1991). Human monoamine oxidase A and B genes exhibit identical exon-intron organization. Proc Natl Acad Sci U S A.

[6] Youdim MB, Edmondson D, Tipton KF (2006). The therapeutic potential of monoamine oxidase inhibitors. Nat Rev Neurosci.

[7] Shih JC, Chen K, Ridd MJ (1999). Monoamine oxidase: from genes to behavior. Annu Rev Neurosci.

[8] Yang X, Shi C, Tong R, Qian W, Zhau HE, Wang R, Zhu G, Cheng J, Yang VW, Cheng T, Henary M, Strekowski L, Chung LW (2010). Near IR heptamethine cyanine dye-mediated cancer imaging. Clin Cancer Res.

[9] Duffy MJ, Maguire TM, Hill A, McDermott E, O'Higgins N (2000). Metalloproteinases: role in breast carcinogenesis, invasion and metastasis. Breast Cancer Res.

[10] Hanahan D, Weinberg RA (2000). The hallmarks of cancer. Cell.

[11] Gordon S (2003). Alternative activation of macrophages. Nat Rev Immunol.

[12] Gong D, Shi W, Yi SJ, Chen H, Groffen J, Heisterkamp N (2012). TGFbeta signaling plays a critical role in promoting alternative macrophage activation. BMC Immunol.

[13] Wu JB, Shao C, Li X, Li Q, Hu P, Shi C, Li Y, Chen YT, Yin F, Liao CP, Stiles BL, Zhau HE, Shih JC, Chung LW (2014). Monoamine oxidase A mediates prostate tumorigenesis and cancer metastasis. J Clin Invest.

[14] Wu JB, Lin TP, Gallagher JD, Kushal S, Chung LW, Zhau HE, Olenyuk BZ, Shih JC (2015). Monoamine oxidase A inhibitor-near-infrared dye conjugate reduces prostate tumor growth. J Am Chem Soc.

[15] Kaina B, Christmann M, Naumann S, Roos WP (2007). MGMT: key node in the battle against genotoxicity, carcinogenicity and apoptosis induced by alkylating agents. DNA Repair (Amst).

[16] Salgado R, Benoy I, Bogers J, Weytjens R, Vermeulen P, Dirix L, Van Marck E (2001). Platelets and vascular endothelial growth factor (VEGF): a morphological and functional study. Angiogenesis.

[17] Gonzalez FJ, Rueda A, Sevilla I, Alonso L, Villarreal V, Torres E, Alba E (2004). Shift in the balance between circulating thrombospondin-1 and vascular endothelial growth factor in cancer patients: relationship to platelet alpha-granule content and primary activation. Int J Biol Markers.

[18] Jhaveri N, Cho H, Torres S, Wang W, Schonthal AH, Petasis NA, Louie SG, Hofman FM, Chen TC (2011). Noscapine inhibits tumor growth in TMZ-resistant gliomas. Cancer Lett.

[19] Chen K, Ou XM, Chen G, Choi SH, Shih JC (2005). R1, a novel repressor of the human monoamine oxidase A. J Biol Chem.

[20] Cho HY, Wang W, Jhaveri N, Torres S, Tseng J, Leong MN, Lee DJ, Goldkorn A, Xu T, Petasis NA, Louie SG, Schonthal AH, Hofman FM, Chen TC (2012). Perillyl alcohol for the treatment of temozolomide-resistant gliomas. Mol Cancer Ther.

