# Supplementary material for: Monoamine oxidase A (MAO A) inhibitors decrease glioma progression
Source: Oncotarget. 2016 Feb 9;7(12):13842–53. doi: 10.18632/oncotarget.7283 (PMC4924682; doi:10.18632/oncotarget.7283)
Supplement: Supplementary file 1 [file oncotarget-07-13842-s001.pdf]

SUPPLEMENTARY MATERIAL AND FIGURE

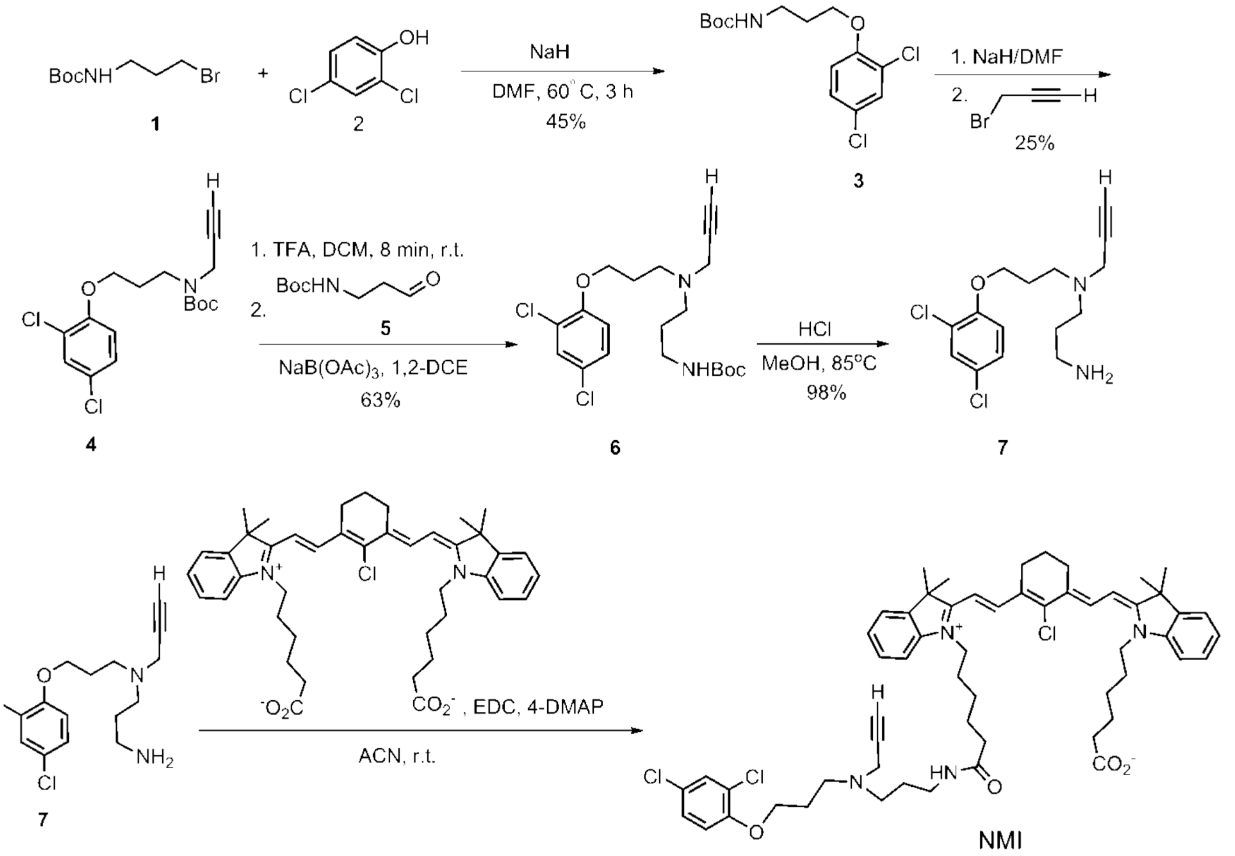

Supplementary Figure S1: Overall scheme for synthesis of NMI.

Detailed method for synthesis of NMI

All reagents and solvents were obtained from commercial sources and were used as received unless otherwise stated. All reactions involving moisture-sensitive reagents were conducted under argon atmosphere with anhydrous solvents and flame-dried glassware. Hygroscopic liquids were transferred via a syringe and were introduced into reaction vessels through rubber septa. Reaction product solutions were concentrated using a rotary evaporator at 30-150 mm Hg. Column chromatography was performed on silica gel (230-400 mesh) using reagent grade solvents. Analytical thin-layer chromatography (TLC) was performed on glass-backed, pre-coated plates (0.25 mm, silica gel 60, F-254, EM Science). Analytical HPLC were performed on Microsorb-MV C_8 reverse-phase column (250 × 4.6 mm, Varian) using Shimadzu LC-10A VP pump and Shimadzu SPD 10A VP UV-vis variable-wavelength detector. Preparative HPLC purifications were carried out with C_8 reverse phase preparative column (Grace Davison). The flow rate for preparative reverse-phase HPLC was 4 mL/min. In all cases, 5% - 95% gradients of acetonitrile in 0.1% aqueous trifluoroacetic acid (TFA) were used as eluents. Water (18 MΩ) was obtained from a Barnstead water purification system, and all buffers were 0.2 μm filtered. Nuclear magnetic resonance (NMR) spectra were collected on instruments in the indicated solvents. The identity and purity of each intermediate and the final product were confirmed by ^{1}H NMR (Varian Mercury 400 MHz) and mass spectrometry (Agilent 6520 time-of-flight system).

t-Butyl (3-bromopropyl)carbamate (1):

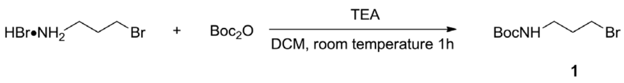

To a 100-mL round-bottom flask equipped with a magnetic stirrer was added 3-bromopropylamine hydrobromide (6.57 g, 30 mmol, 1.0 eq.) and

dichloromethane (160 mL). To the resultant solution was added di-tert-butyl dicarbonate (6.54 g, 30 mmol, 1.0 eq.) in dichloromethane (110 mL), followed by triethylamine (4.8 mL, 34.5 mmol, 1.15 eq.). The solution was stirred at room temperature for 95 minutes. The reaction was washed twice with saturated sodium bicarbonate (70 mL, 40 mL) and once with saturated sodium chloride (100 mL). The organic phase was dried over sodium sulfate, filtered, and solvent removed *in vacuo* to yield **1** (6.91 g, 97% yield). ^{1}H NMR (400 MHz, $CDCl_3$) δ: 4.67 (bs, 1 H), 3.43 (t, *J*= 6 Hz, 2H), 3.26 (m, 2 H), 2.04 (m, 2 H), 1.43 (s, 9 H).

t-Butyl 3-(2,4-dichlorophenoxy) propylcarbamate (3):

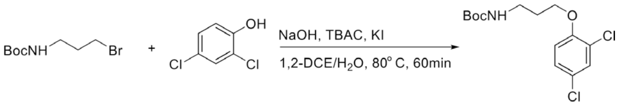

1 2 3

To a 20-mL scintillation vial equipped with a magnetic stirrer was added 2,4-dichlorophenol (**2**, 237 mg, 1.45 mmol, 1.0 eq.) followed by 1,2-dichloroethane (5 mL). To the resultant solution was added **1** (346 mg, 1.45 mmol, 1.0 eq.), tetrabutylammonium chloride (44.2 mg, 0.159 mmol, 0.11 eq.), followed by potassium iodide (24.1 mg, 0.145 mmol, 1.0 eq). NaOH (5 mL of 10%) was added and the mixture was stirred at 80°C for 1h. The biphasic mixture was partitioned and the aqueous layer was extracted twice with DCM (7 mL). The organic phases were pooled, dried over magnesium sulfate, filtered, and then concentrated. The material (512.7 mg) was then columned over silica gel (2.5” × 1”, height × width) eluting with 100 mL 10% ethyl acetate in hexanes, 100 mL 15% ethyl acetate in hexanes, and 100 mL 25% ethyl acetate. Product**3** elutes in 15% ethyl acetate fractions. Yield 310 mg (67%). ^{1}H NMR (400 MHz, CDCl3) δ: 7.32 (d, *J* = 2.4 Hz, 1 H), 7.14 (dd, J_1 = 8.8 Hz, J_2 = 2.4, 1 H), 6.80 (d, J_1 = 5.2 Hz, 1 H), 5.18 (bs, 1 H), 4.04 (t, J = 6 Hz, 2 H), 3.34 (m, 2 H), 2.00 (m, 2 H), 1.41 (s, 9 H).

t-Butyl 3-(2,4-dichlorophenoxy) propyl(prop-2-ynyl)carbamate (4):

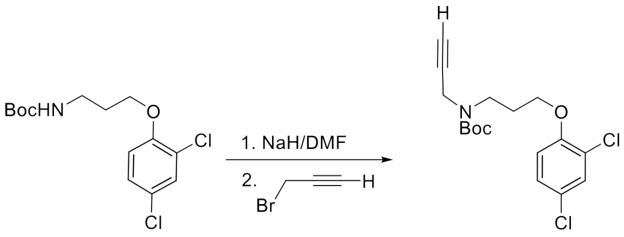

3 4

To a 20-mL scintillation vial was added **3** (500 mg, 1.57mmol, 1.0 eq) followed by sodium hydride (63 mg, 1.57 mmol, 1.0 eq.). The resulting solution was stirred for 15min with venting to atmosphere, followed by the addition of propargyl bromide (470 mL of 80% solution in toluene (w/v), 3.14 mmol, 2.0 eq.). Note: the solution changes color from yellowish to brown upon addition of propargyl bromide. The reaction was stirred at room temperature for 4 h and solvent was removed via an airstream and the residue was dried *in vacuo*. The residue was reconstituted in 5 mL DCM and 1.65g of celite-545 was added. The mixture was then evaporated to dryness. This material was loaded onto a 5” × 1” (height × width) plug of SiO_2 equilibrated with hexanes and product was eluted with 100 mL 2% ethyl acetate in hexanes, 200 mL 5% ethyl acetate in hexanes, and 200 mL 30% ethyl acetate in hexanes. Fractions containing product were pooled (product elutes between 5% and 30% ethyl acetate in hexanes). Yield 292.6 mg of **4** (52% yield) ^{1}H NMR (400 MHz, CDCl3) δ: 7.35 (d, *J* = 2.8 Hz, 1 H), 7.16 (dd, J_1 = 9.2 Hz, J_2 = 2.8, 1 H), 6.82 (d, J_1 = 8.8 Hz, 1 H), 5.18 (bs, 1 H), 4.05 (m, 4 H), 3.55 (t, *J*= 7.2 Hz, 2 H), 2.18 (t, *J* = 2.4 Hz, 2 H), 2.00 (m, 2 H), 1.43 (s, 9 H).

N-(3-(2,4-dichlorophenoxy) propyl)prop-2-yn-1-aminium trifluoroacetate:

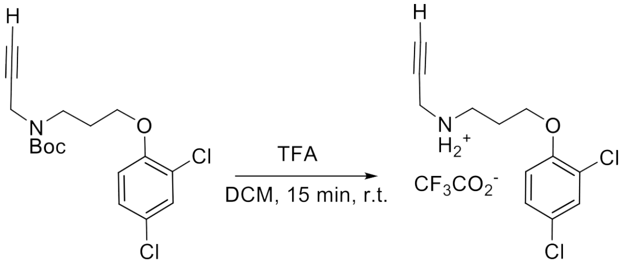

4

To a solution of *tert*-butyl 3-(2,4-dichlorophenoxy) propyl(prop-2-ynyl)carbamate **4** (147.6 mg, 412 µmol) in 4 mL DCM in a 20 mL-scintillation vial equipped with a stir bar was added 1 mL TFA at room temperature while stirring. After 15 min, HPLC (5-95% B (A=0.05% aqueous TFA; B=acetonitrile) over 20 min, 0.8 mL/min) indicated completion of the reaction. The solvents were removed under reduced pressure. The residue was co-evaporated with ACN times and then used in the next step without further purification.

t-Butyl (3-oxopropyl)carbamate (5):

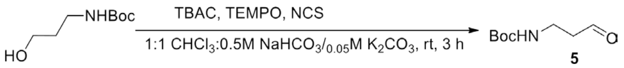

5

To a 100 mL round bottom flask was added *t*-butyl (3-hydroxypropyl)carbamate(1.8 g, 10.3 mmol, 1.0 eq.) followed by 35 mL of $CHCl_3$ and 35 mL of 0.5M $NaHCO_3$/0.05M K_2CO_3. To this mixture was added

tetrabutylammonium chloride (288 mg, 1.04 mmol, 0.1 eq.), TEMPO (162 mg, 1.04 mmol, 0.1 eq.), and N-chlorosuccinimide (2.1 g, 15.7 mmol, 1.53 eq.) with vigorous stirring. After three hours the phases were separated, the organic layer was dried, and solvent was removed to give a viscous orange oil. This material was subjected to column chromatography over silica gel using a 4” × 1” (height × width) plug of SiO_2 and eluting with 75 mL hexanes, 100 mL 20% ethyl acetate in hexanes, 100 mL 30% ethyl acetate in hexanes, 50 mL ethyl acetate. Yield 732.8 mg (41%).

t-Butyl (3-((3-(2,4-dichlorophenoxy)propyl)(prop-2-yn-1-yl)amino)propyl)carbamate (6):

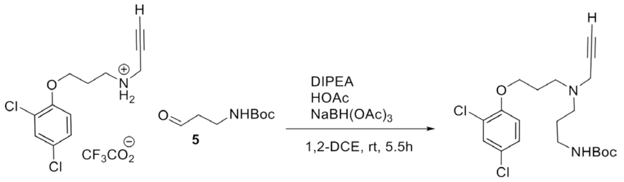

4 **5** **6**

To 100-mL round bottom flask equipped with a stir bar was added *N*-(3-(2,4-dichlorophenoxy)propyl) prop-2-yn-1-aminium trifluoroacetate **4** (1.25 g, 3.36 mmol, 1.0 eq.) in 25 mL 1,2-DCE . Next, **5** (0.641 mg, 3.4 mmol, 1.1 eq.) was added followed by DIPEA (584 mL, 3.36 mmol, 1.0 eq), acetic acid (330 mL, 5.81 mmol, 1.73 eq.), and sodium triacetoxyborohydride (1.14 g, 5.38 mmol, 1.6 eq.). The reaction was stirred for 5.5 h until approximately 85% conversion was reached, at which point the organic layer was washed twice with sodium bicarbonate (100 mL), dried over anhydrous $MgSO_4$, filtered, and solvent was removed *in vacuo* to give 1.12 g of crude **6**. This material was used in the next step without further purification.

N1-(3-(2,4-dichlorophenoxy)propyl)-N1-(prop-2-yn-1-yl)propane-1,3-diaminium 2,2,2-trifluoroacetate (7)

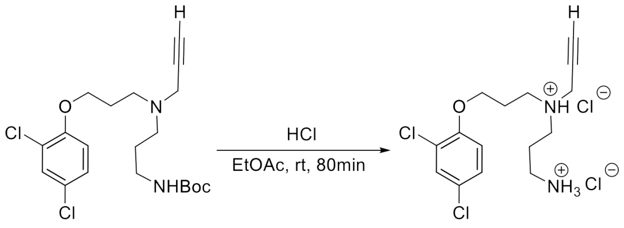

6 **7**

A solution of *N*-(3-(2,4-dichlorophenoxy)propyl)-*N*-(prop-2-yn-1-yl)propane-1,3-diaminium chloride **7** (16 mg, 38.5 µmol) in 10 mL ethyl acetate containing 248 mg of HCl was stirred mechanically. The resulting reaction was stirred at room temperature for 80 min before reaction completion was confirmed by LC/MS. The product was evaporated under reduced pressure and used in the next step without further purification.

NMI

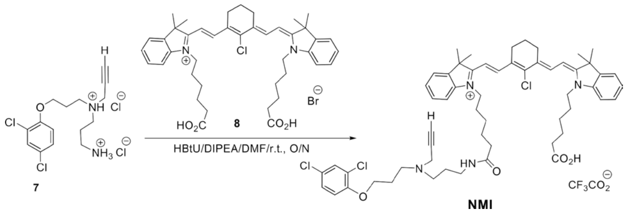

7 **8** **NMI**

To a 20-mL scintillation vial was added MHI-148 dye **8** (5.79 mg, 8.47 mmol) followed by DMF (500 mL), DIPEA (1.1 mL, 8.47 mmol, 1.0 eq.), and HBtU (3.2 mg, 8.47 mmol, 1.0 eq.). The mixture was stirred for 10 min at room temperature after which time **7** (4.6 mg, 8.47 mmol, 1.0 eq.) and DIPEA (2.2 mL, 16.94 mmol, 2.0 eq.) in DMF (500 µL) were added. The reaction was covered in foil, and stirred overnight at room temperature. The DMF was evaporated by airstream and the residue was purified on preparative silica gel plates using DCM/isopropanol 1:1 as an eluent. Yield of **NMI** 1.2 mg (14%). ^{1}H NMR (400 MHz, $CDCl_3$): δ 1.49 (m, 2H, γ-$C\underline{H}_2$(COOH)), 1.56 (m, 2H, γ-$C\underline{H}_2$(CONHCH$_2$-)), 1.67 (s, 6H,$C\underline{H}_3$), 1.71 (s, 6H,$C\underline{H}_3$), 1.80 (m, 2H, β-$C\underline{H}_2$), 1.82 (m, 2H, β-$C\underline{H}_2$), 1.86 (m, 2H, δ-$C\underline{H}_2$), 1.88 (m, 2H, δ-$C\underline{H}_2$), 1.94 (s, 2H, O-CH$_2$$C\underline{H}_2CH_2$N-), 1.97 (s, 2H, NH-CH$_2$$C\underline{H}_2CH_2$N-), 2.09 (s, 2H, CH$_2$), 2.56-2.57 (m, 4H, α-CH$_2$), 2.68-2.70 (m, 4H, $C\underline{H}_2$N$C\underline{H}_2$), 2.71 (s, 2H, CH$_2$C=), 2.75 (s, 2H, CH$_2$C=), 3.38 (m, 2H, -NHC$\underline{H}_2$), 4.04 (t, 1H, $\underline{H}$CΞ$\underline{C}$-), 4.05 (t, 4H, N-C$\underline{H}_2$), 4.05 (t, 2H, O-C$\underline{H}_2$), 4.07 (t, 2H, CH$_2$CΞ), 4.57 (bs, 1H, N$\underline{H}$-CH$_2$CH$_2$), 6.04 (d, 1H, C$\underline{H}$=CH), 6.32 (d, 1H, C$\underline{H}$=CH), 6.85 (d, 1H, Ar-$\underline{H}$), 7.05 (d, 1H, Ar-$\underline{H}$), 7.17 – 7.42 (m, 9H, Ar-$\underline{H}$), 8.28 (d, 1H, CH=C$\underline{H}$), 8.40 (d, 1H, CH=C$\underline{H}$). HRMS calcd. for $C_{57}H_{69}Cl_3N_3O_4S$ $C_{57}H_{70}Cl_3N_4O_4$ *m/z* 979.4457; observed *m/z* 979.4463. Please note that the NMI structure in this paper represents the linkage between clorgyline and MHI and it is an amide bond.
